# Human head and neck cancer cell lines response to cold atmospheric plasma activated media is affected by the chemistry of culture media

**DOI:** 10.1016/j.heliyon.2024.e41458

**Published:** 2024-12-25

**Authors:** Viviana di Giacomo, Marwa Balaha, Asia Pece, Ilaria Cela, Gianluca Fulgenzi, Giovanna Orsini, Tatiana Spadoni, Tirtha Raj Acharya, Nagendra Kumar Kaushik, Eun Ha Choi, Monica Rapino, Mariangela Mazzone, Gabriella Mincione, Gianluca Sala, Eloisa Sardella, Vittoria Perrotti

**Affiliations:** a Department of Pharmacy, “G. d’Annunzio” University of Chieti-Pescara, Chieti, Italy; b UdA-TechLab, Research Center, “G. d’Annunzio” University of Chieti-Pescara, Chieti, Italy; c Department of Pharmaceutical Chemistry, Faculty of Pharmacy, Kafrelsheikh University, 33516, Kafr El Sheikh, Egypt; d Department of Innovative Technologies in Medicine & Dentistry, “G. d’Annunzio” University of Chieti-Pescara, Chieti, Italy; e Center for Advanced Studies and Technology (CAST), “G. d’Annunzio” University of Chieti-Pescara, Chieti, Italy; f Department of Clinical and Molecular Sciences, Polytechnic University of Marche, Ancona, Italy; g Department of Clinical Sciences and Stomatology (DISCO), Polytechnic University of Marche, Ancona, Italy; h Plasma Bioscience Research Center, Department of Electrical and Biological Physics, Kwangwoon University, Seoul, 01897, South Korea; i Genetic Molecular Institute of CNR, Unit of Chieti, “G. d’Annunzio” University of Chieti-Pescara, Chieti, Italy; j CNR- Istituto di Nanotecnologia (CNR-NANOTEC) UoS Bari, c/o Dipartimento di Chimica, Università degli Studi di Bari Aldo Moro, via Orabona, 4, 70126, Bari, Italy

**Keywords:** Cold atmospheric plasma, Plasma activated media, Apoptosis, Cell cycle, Cell proliferation, Head and neck cancer, Reactive oxygen species, Hydrogen peroxide

## Abstract

Survival rate of head and neck squamous cell carcinomas (HNSCC) patients are still to date very poor, and the application of innovative clinical approaches are urgently needed. Cold atmospheric plasmas (CAPs) are partially ionized gases that have shown anti-tumor effectiveness over a wide range of cancer types with potential application into clinics. However, the comprehension of the mechanisms underlying indirect CAP effects plays a key role for the prediction of treatment outcomes. In our work, we assessed the potential application of indirect CAP, by using plasma activated media (PAM) and plasma-treated liquids (PTL), as therapeutic strategies for HNSCC treatment. The impact of PAM obtained from exposure to CAP for different times was evaluated in three head and neck cancer (HNC) cell lines (HSC3, FaDu, CAL-27). Cytotoxic effects as inhibition of proliferation, apoptosis rate and cell cycle modifications were tested for the different PAM, showing a time- and cell culture media-dependence tightly related to the chemical composition of PAM considered. In addition, cytotoxic effects were also observed on HNC, two bladder cancer models and one breast cancer cell line when considering PTL, paving the way for their application into a clinical setting.

## 1 Introduction

Head and neck cancers (HNCs) encompass a diverse group of malignancies originating from the oral cavity, pharynx, larynx, and related structures, wherein squamous cell carcinomas (HNSCC) accounting for approximately 90 % of cases [1]. Major risk factors include smoking, alcohol consumption, and HPV infection [2]. The incidence of HNSCC continues to rise, with nearly 700,000 new cases reported annually [3], which makes HNCs the seventh most prevalent cancer globally [3]. Tragically, more than half of HNSCC are diagnosed at a locally advanced stage. Despite advancements in therapeutic strategies, the 5-year survival rate for HNSCC remains alarmingly low at 40–50 %, largely due to late-stage diagnosis, recurrent metastasis, therapy resistance, and relapse [1,4,5]. The current standard of care involves surgical resection, often supplemented by radiotherapy and chemotherapy; however, these treatments are associated with significant morbidity, diminished quality of life, and limited improvement in long-term outcomes [6]. In this context, emerging therapeutic approaches such as cold atmospheric plasmas (CAPs), have gained considerable attention for their potential for as targeted and minimally invasive cancer treatments [7].

CAPs are partially ionized gases generated under non-equilibrium thermodynamics conditions, typically close to room temperature. They consist of atoms and molecules in various energy states, electrons, ions, reactive oxygen and nitrogen species (RONS), as well as ultraviolet–visible (UV–vis) radiation and an electric field [8]. CAP has demonstrated notable a anticancer effects across a wide range of cancer models, including carcinomas [9], melanomas [10], neuroectodermal [11] and hematopoietic malignancies [12]. CAP application can be broadly categorized into two distinct approaches: direct irradiation of cells or tissues, and indirect application through plasma-treated liquids (PTL), such as plasma activated cell culture media (PAM) or plasma treated hydrogels (PTH) [[13], [14], [15]]. Recently, the indirect approach has garnered increased attention due to its versatility and potential for broader applications [13,[16], [17], [18], [19]].

The clinical application of CAP has recently emerged as a very active area of research [20,21]. Early evidence in the treatment of locally advanced HNCs has shown promising results, including improved quality of life, reduced odor, and decreased reliance on pain medications [[22], [23], [24]]. Partial remission has been observed in certain patients along with an increased in apoptotic cells in tissues exposed to CAP [25]. In general, indirect treatments have demonstrated comparable effectiveness to direct CAP applications, with the added advantage that PTL can be injected into the body, thereby reaching tissues that are inaccessible through direct irradiation. This approach also allows for controlled, long-term treatment of cancer cells [13,19,26]. Despite these encouraging outcomes, the mechanisms underlying CAP's selective cytotoxicity remain incompletely understood and have not yet been definitely validated [27]. One key hypothesis is that CAP-generated RONS act as the primary effectors. While low to moderate levels of RONS serve as signaling molecules in cellular processes [28], elevated levels have been associated with cellular damage, leading to cell cycle arrest and apoptotic or necrotic cell death [29]. Cancer cells exhibit higher metabolic activity than healthy cells, resulting in increased intrinsic RONS production. As a result, administering PAM with low levels of exogenous RONS can push cancer cells beyond their toxicity threshold [30]. The anti-cancer effects of PAM are tought to be primarly driven by the formation of stable RONS within the media [31,32] and some studies demonstrated their anticancer effects by administering PAM to animal models [33,34].

Nevertheless, both *in vivo* and *in vitro* investigations demonstrated that not all cells respond equally to CAP treatment or PAM [14] and the cause - effect relationship between RONS and specific cellular outcomes has yet to be fully elucidated [35].

While PTL offer clear advantages over direct plasma treatment for internal tumours, the concentration of reactive species generated in cell culture media varies significantly across different cell types, making it difficult to precisely define and complicating the ability to draw definitive conclusions [36,37]. Understanding the chemical composition of these species is critical for customizing plasma properties for disease-specific applications, facilitating addressing a faster clinical approval [38].

RONS can be classified into short-lived or long-lived reactive species, based on their stability. Due to their higher stability, long-lived reactive species - primarly hydrogen peroxide, nitrites, nitrates, peroxynitrites - are considered the key biologically relevant components in PAM treatments [39]. Among these, H_2_O_2_, a stable and diffusible ROS, has been implicated in modulating redox-sensitive pathways and contributing to apoptotic responses in cancer cells [31,40]. Reactive species, which are generated not only by external sources such as CAP, but are also naturally by-products of cellular metabolism, triggers a defense system in cells to counteract oxidative stress. This system aims to prevent DNA damage and apoptosis [41,42]. However, when overwhelmed by CAP-generated reactive species, the capacity of this defence system becomes insufficient, ultimately leading to cell death [41]. Preliminary *in vitro* and *in vivo* investigations have shown that both CAP and PAM selectively reduced the viability of HNCs cells, while normal control cells remain unaffected [7,13,[43], [44], [45], [46], [47], [48]].

To this end, understanding the main cellular response pathways, the stability of RONS in different PAM formulations, and comparing the response of malignant and non-malignant cells would improve treatment prediction and represent a critical step in advancing this innovative technology toward clinical application. Recently, our laboratory developed a novel ion chromatography-based approach to chemically characterize PAM, aiming to standardize their chemical analysis [49].

The current study aims to investigate the response of a panel of human HNC cell lines (FaDu, HSC3, CAL-27) to PAM, with a particular emphasis on understanding the impact of stable RONS in PAM on the observed effects. The study specifically focuses on total ROS content and employs a comprehensive methodology to provide a mechanistic understanding of the cytotoxic effects, particularly in terms of proliferation inhibition, apoptosis induction, and cell cycle regulation induced by CAP-activated media on HNC cells, thereby shedding light on the potential of CAP as a therapeutic strategy.

## 2 Materials and methods

### 2.1 CAP jet configuration and its diagnosis techniques

**Fig. 1:**
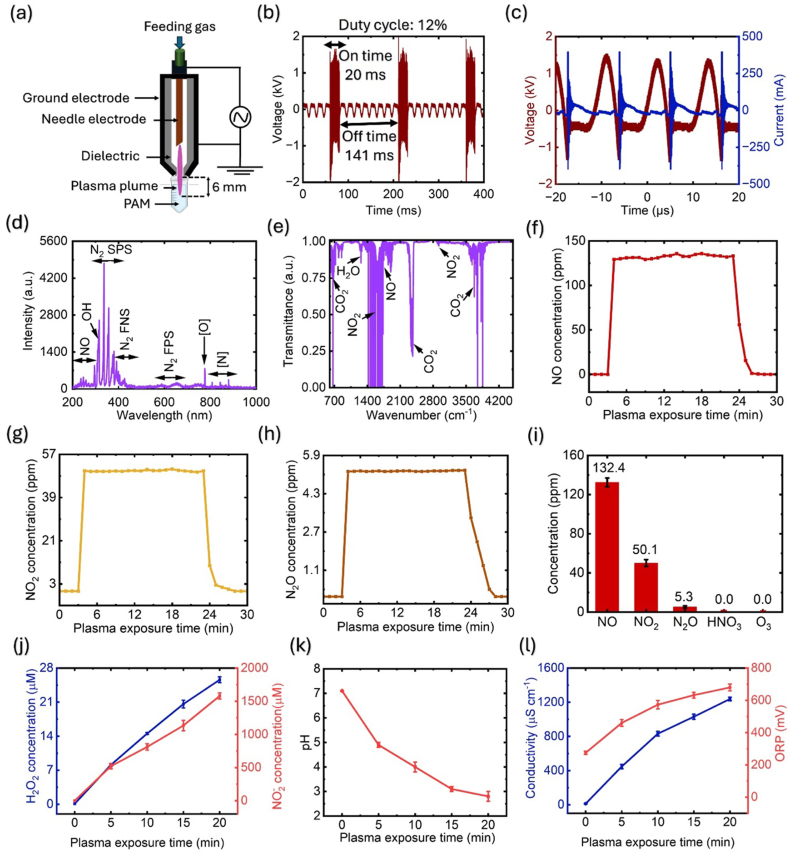
Plasma jet configuration and diagnosis. (a) Schematic diagram of cold atmospheric plasma (CAP) jet, (b–c) current-voltage profile, (d) optical emission spectra showing various RONS present in plasma phase, (e–h) Fourier transform infrared spectra and quantification of the various plasma generated species in gas phase such as NO, NO_2_, N_2_O, HNO_3_ and O_3_, (i) time average concentration of RONS produced in the gas phase, (j) quantification of H_2_O_2_ and NO_2_^−^ present in plasma-activated water (PAW), (k) pH measurement of PAW and (l) measurement of electrical conductivity and oxidation-reduction potential (ORP) in PAW.

Fig. 1a shows a schematic of the CAP jet used in this study. To ensure smooth plasma generation, the airflow rate was maintained at 2.0 L per min (lpm), with the primary voltage regulated by a voltage controller. A stainless-steel injection needle with a diameter of 1.2 mm and a thickness of 0.3 mm, served as the high-voltage electrode. The outer electrode, made of stainless steel, acted as the ground electrode. This ground electrode had a thickness of 0.3 mm, a length of 6.0 mm, and a centrally perforated hole of 0.7 mm to facilitate continuous plasma plume generation. The distance between the tip of the CAP jet tip and the precursor was set to 6.0 mm, with a discharge gap of 2.0 mm between the inner and outer electrodes. All experiments were conducted in a closed chamber at a relative humidity of (60 ± 5) % and a room temperature of (300 ± 4) K. The media volumes of 2 mL and 5 mL were treated with the CAP jet for durations ranging from 5 to 20 min. Plasma diagnostics for this specific setup and conditions have already been comprehensively documented in our prior work by di Giacomo et al. [49]. These previous diagnostics included measurements of electron temperature, gas bulk temperature, rotational and vibrational temperatures, electron density, and nitrogen metastable state density under identical plasma and device conditions. This study mainly focuses on applying those established parameters to assess treatment effects under consistent conditions, specifically examining how the response of human HNC cell lines to PAM is influenced by the chemistry of the culture media used.

The current-voltage characteristics were analysed using a digital oscilloscope (WaveSurfer 434) equipped with HV and current probes. The optical emission spectra of CAP jet-induced plasma were analysed using optical emission spectroscopy (OES) (HR4000, Ocean Optics). Real-time gas-phase RONS concentrations generated by the CAP jet were measured using Fourier transform infrared spectroscopy (FTIR) with the MATRIX-MG5 automated FTIR gas analyser and OPUS GA software. This system continuously analysed the plasma composition, including various RONS such as NO, NO₂, N₂O, HNO₃, and O₃, by connecting the gas outlet of the CAP jet to the analyser. The concentration of H₂O₂ in de-ionized water (DW) after CAP jet treatment was determined using the QuantiChrom™ peroxide assay kit that based on the chromogenic Fe³⁺-xylenol orange reaction with the absorbance measured at 585 nm. The detailed experimental procedure is throughly described in a recently published research article [50]. The concentration of NO₂⁻ was determined by using QuantiChrom™ nitric oxide assay kit by the modified Griess method, which measured absorbance at 540 nm as outlined in the recently published research work [50]. Additionally, the pH, electrical conductivity, and oxidation-reduction potential (ORP) of the plasma-activated water (PAW) were measured using an electrochemical meter [PHS-3E, Shanghai INESA Scientific Instrument Co. Ltd., Shanghai China].

### 2.2 Cell lines and treatments

Human gingival fibroblasts (hGF) were purchased from CLS (#300703; GmbH) and cultured in DMEM/F12 (Gibco, Waltham, MA, USA) supplemented with heat-inactivated 5 % FBS, 10 mM HEPES, 100 units/mL penicillin, and 100 μg/mL streptomycin (Sigma-Aldrich). FaDu (from human hypopharyngeal carcinoma) and TCCSUP (from human urinary bladder carcinoma) cell lines were purchased from American Type Culture Collection (ATCC #HTB-43 and #TCP-1020, Rockville, MD, USA), and grown in EMEM supplemented with 1 % Non-Essential Amino Acids (Gibco, Waltham, MA, USA). HSC3, HOC621 and CAL-27 (from human tongue squamous carcinoma) cell lines were a kind gift by prof. Lorenzo Lo Muzio (University of Foggia, Foggia, Italy) and were cultured in RPMI (HSC3) and DMEM (HOC621 and CAL-27) (Gibco, Waltham, MA, USA). T24 (from human urinary bladder carcinoma) and MDA MB361 (from human breast carcinoma) cell lines were purchased from ATCC (#TCP-1020 and #HTB-27, Rockville, MD, USA) and cultured in McCoy's 5A Medium and DMEM, respectively (Gibco, Waltham, MA, USA). DMEM/F12, RPMI, EMEM, McCoy's 5A Medium and DMEM culture media were supplemented with 10 % heat-inactivated fetal bovine serum (FBS; Gibco) and 1 % penicillin/streptomycin (Gibco). All cell lines were cultivated at most for 1 month and maintained at 37 °C in humified air with 5 % CO_2_.

For CAP indirect treatments, PAM were generated by exposing serum-free media to a the plasma jet previously described [51]. Briefly, the working distance between the capillary of the plasma device and the liquid medium surface was set to 6 mm. Two different media volumes - 2 mL and 5 mL - were exposed to CAP for 5, 10, and 20 min. Immediately after activation, 10 % heat-inactivated FBS was added to the PAM to match the growth conditions required for cell treatment. Treatments were performed by incubating cells with the distinct PAM for 24, 48 and 72 h.

Doxorubicin (#D5220, CliniSciences, Guidonia Montecelio, Roma, Italy) treatments, used as positive control for cell proliferation inhibition, were carried out in complete medium at a final concentration of 1 μM.

To assess the behaviour of cells exposed to a PTL not derived from a cell culture medium, a clinically approved Electrolyte Rehydrating III solution (SIII, Fresenius Kabi) was exposed to an experimental planar volume dielectric barrier discharge (DBD) system designed by CNR-NANOTEC. The SIII solution is composed by sodium, potassium, calcium, magnesium chlorides along with sodium acetate and sodium citrate. A glass chamber (150 mm dia.) underneath the plasma source was filled with 60 mL of liquid. The liquid was remotely treated by plasma at a distance of 2 mm from the plasma zone. The liquid was exposed to a volume discharge as wide as the HV copper electrode (76 mm dia, 0.5 mm thick) and was treated with the plasma effluents, which diffused through a ground mesh 2 mm away from the liquid surface. The HV copper electrode was connected to a microsecond‐pulsed generator (Alma-Pulse; AlmaPlasma s.r.l.) operating at a field frequency of 9 kHz, with an applied voltage of 10 kV, and pulsed at a duty cycle (D.C.) of 25 % (i.e.,25 ms plasma on, t_on_) over a period of 100 ms (t = t_on_ + t_off_). Before igniting the plasma, the gap between the liquid and the discharge was purged with the gas feed for 2 min. All discharges were ignited at a constant gas flow rate of 1 L min^−1^. The treatment times varied from 5 to 20 min. Pure synthetic air (air liquid, 99.999 %) was used as gas feed of the DBD source. To enhance the production of H_2_O_2_, SIII was supplemented with 300 mg/L of L-tyrosin [52] (SIII-Tyr) and exposed to air CAP treatment for 5, 10 and 20 min.

### 2.3 Cell proliferation assay

Cell proliferation was assessed using the MTS [3-(4,5-dimethylthiazol-2-yl)-5-(3-carboxymethoxyphenyl)-2-(4-sulfophenyl)-2H-tetrazolium] assay (#G3581, Promega, Madison WI, USA). Briefly, cells were seeded and grown under standard culture conditions in 96-well plates at a density of 5 x 10^3^ cells per well, one day prior to treatment. After 24 h, 0.1 mL per well of the distinct PAM or plasma treated SIII supplemented with tyrosine, or 1 μM doxorubicin was added to the cells and further incubated. At 24, 48, and 72 h post-treatment, MTS solution was added to each well at a final concentration of 10 % (v/v) and incubated for 2 h at 37 °C. Cell proliferation was evaluated by measuring the colorimetric absorbance at 490 nm using a multi-plate reader (Tecan trading, Männedorf, Switzerland). Cell proliferation inhibition in for each treatment is was expressed as a percentage of the treated cell number relative to the corresponding untreated control cells at 24, 48, and 72 h and was calculated as follows:

% Inhibition = (100 − (OD_T__est_/ OD_non-tre__at__ed_)) x 100

Three independent experiments were conducted, each performed in technical triplicate.

### 2.4 Quantification of total ROS

Due to the complexity and diversity in the chemical composition of the liquids used in this paper, the 2′, 7′-Dichlorodihydrofluorescein diacetate (DCFH-DA) method was employed to detect general reactive oxygen species (ROS) production. This method is one of the fastest, easiest, most user-friendly, and accessible techniques for monitoring ROS generation. It is based on the detection of ROS-sensitive fluorescent probes by a fluorescence microplate reader. When PTL are exposed to DCF-HA, the probe is oxidized to a DCF radical, which is then further oxidized by molecular oxygen to form DCF, the fluorescent product. DCF is maximally excited at 485 nm and emitting at 528 nm. ROS levels were quantified after exposing two distinct volumes – 2 mL and 5 mL – of RPMI, EMEM, and DMEM culture media or SIII supplemented with tyrosine to CAP for 5, 10, and 20 min. In a black 96-well plate (Corning, New York, USA), 150 μL of each PAM or PTL was mixed with 1 μL of 2 mM DCFH-DA (Toronto Research Chemicals, Toronto, Canada) and incubated for 30 min at 37 °C. Untreated culture media or SIII-Tyr solution served as controls. Total ROS quantification for PTL and PAM was conducted at baseline (t = 0), and after 24, 48, and 72 h of incubation at 37 °C and 5 % CO_2_, by measuring fluorescence at 485/528 nm using a Synergy H1 Hybrid microplate reader (BioTek®, Santa Clara, CA, USA). The results were expressed as the percentage of fluorescence relative to control.

### 2.5 Flow cytometry apoptosis detection

Apoptosis was assessed 24 h after incubation of the above-mentioned cell lines with 2 mL PAM treated for 5 min and 5 mL PAM treated for 5, 10, and 20 min, using a FITC Annexin-V apoptosis detection kit (BD Pharmingen, San Diego, CA, USA), following the manufacturer's instructions. Briefly, 1 x 10^5^ cells were gently re-suspended in 100 μL of 1X binding buffer and incubated for 15 min at room temperature in the dark with 5 μL of Annexin-V-FITC and 5 μL of Propidium Iodide. After the addition of 200 μL of binding buffer, samples were analysed with a Cytoflex flow cytometer with the FL1 and FL3 detector in a log mode, using the Cytoexpert analysis software (both from Beckmann Coulter, Brea, California, USA). For each sample, at least 5000 events were collected. Viable cells were identified as Annexin-V^neg^/PI^neg^ while apoptotic cells were identified as Annexin-V^pos^.

### 2.6 Cell cycle analysis

Cell cycle analysis was conducted 24 h after incubation of the cell lines mentioned above with 2 mL of PAM treated for 5 min and 5 mL of PAM treated for 5, 10, and 20 min. Approximately 3 × 10^5^ cells per experimental condition were harvested, fixed in 70 % cold ethanol, and stored at 4 °C overnight. The cells were then resuspended in 5 μg/ml PI and 100 μg/ml RNAse. Cell cycle profiles (at least 5000 events) were analysed by using a CytoFlex flow cytometer with the FL3 detector in a linear mode and data were processed using the CytoExpert analysis software (both from Beckmann Coulter, Milano, Italy). Data analysis was conducted with ModFit software (Verity Software House, ME, USA).

### 2.7 Transmission electron microscopy (TEM)

For ultrastructural analyses, cells grown on 24-well plates were fixed in 2.5 % glutaraldehyde in 0.1 M cacodylate buffer (pH 7.4), detached from the substrate, centrifuged and embedded in low-melting-point agar. The pellet was trimmed and post-fixed in Osmium Tetroxide 0.5 %, dehydrated through acetone series and flat embedded in epoxy resin using a polystyrene capsule. Ultrathin sections (∼50 nm) were obtained using an MT-X ultratome (RMC; Tucson USA). Ultrastructural characterization was performed on all samples using a CM10 Philips transmission electron microscope (FEI-Philips, USA).

### 2.8 Statistical analysis

The data are presented as the mean ± SD of three independent experiments. Cell proliferation, apoptosis and cell cycle data were analysed using two-way ANOVA test with a p-value of *p* < 0.05 considered statistically significant. Total ROS data were analysed using a *t*-test and a p-value of *p* < 0.05 considered statistically significant.

## 3 Results

### 3.1 Plasma source configuration and diagnosis

Fig. 1a depicts the soft jet plasma setup, whilst Fig. 1b shows the voltage characterization used for the plasma discharge process. A 12 % duty cycle was imposed for the burst discharge plasma mode, with a plasma on time of 20 ms and a plasma off time of 141 ms. Fig. 1c illustrates the current-voltage characterization for the plasma generation process revealing a peak current of 396 mA and a peak voltage of 1.4 kV. OES allows key plasma parameters to be assessed, including electron temperature, vibrational and rotational temperatures, nitrogen metastable state density, and electron density – parameters essential for understanding the plasma dynamics [53]. The CAP jet plasma frequency and dissipated power were determined to be 88 kHz and 1.2 W, respectively. OES analysis (Fig. 1d) identified various RONS, such as ^−^OH radical, N₂ second positive system, N₂ first negative system, N₂ first positive system, and atomic oxygen and nitrogen. Fig. 1(e–h) display the FTIR spectra and quantification of gas-phase RONS, including NO, NO₂, N₂O, HNO₃, and O₃, for CAP jet-generated gases across wavelengths of 700–4200 nm. Fig. 1i highlights the time-averaged concentration of RONS in the gas phase after 20 min of optimized CAP jet operation. Concentrations of NO, NO₂, and N₂O were measured 132.4, 50.1, and 5.3 ppm, respectively, however, HNO₃ and O₃ species were not detected. The concentrations of H₂O₂ and NO₂⁻ in PAW are shown in Fig. 1j after 20 min of the CAP jet treatment using 5 mL of deionized water (DW). The respective concentrations were 25.6 μM and 1580 μM. The CAP jet plasma primarily generates reactive nitrogen species (RNS) such as NO and NO, which dissolve in DW and convert to NO₂⁻ [54]. In contrast, H₂O₂ is less abundant because the CAP jet conditions favor RNS production over ROS. Moreover, H₂O₂ decomposes rapidly in the presence of other reactive species, such as NO and NO₂, further lowering its concentration in PAW. The pH dynamics of DW treated with the CAP jet are shown in (Fig. 1k. The pH decreased as plasma exposure time increased, with a final pH of 2.8 after 20 min of plasma exposure starting from an initial pH of 7.1. The reduction in pH is attributed to the generation of acidic species such as NO₂⁻ and HNO₃, which dissolve into DW [55]. Fig. 1l demonstrates changes in electrical conductivity and ORP of DW as plasma exposure time increased in plasma exposure time from 0 to 20 min. After 20 min, electrical conductivity and ORP reached 1238 μS cm⁻^1^ and 680 mV, respectively. The increase in electrical conductivity is due to the higher ion concentration produced by plasma treatment, while the increase in ORP is linked to elevated levels reactive species generated by the CAP jet, enhancing the oxidative capacity of DW [56].

### 3.2 PAM differentially inhibit cancer cell proliferation

**Fig. 2:**
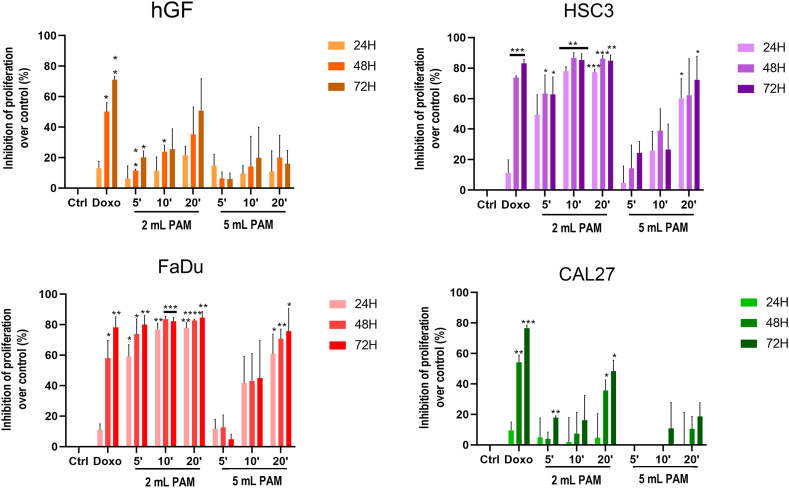
Effects of Plasma-Activated Media (PAM) on (a) normal human gingival fibroblasts (hGF) and on three head and neck squamous cell carcinoma (HNSCC) cell lines (b) HSC3, (c) FaDu, and (d) CAL-27. Histograms represent the percentage of proliferation inhibition over the corresponding control cells (Ctrl, expressed as 0 % of inhibition) at 24, 48, and 72 h. Doxorubicin (Doxo) 1 μM treatment was used as positive control. ∗*p* < 0.05, ∗∗*p* < 0.01, ∗∗∗*p* < 0.001, ∗∗∗∗*p* < 0.0001 *vs* Ctrl same experimental time, refer to statistical significance analysed with two-way ANOVA test.

Fig. 2 shows the results of the MTS assay performed on normal hGF and tumour cells (FaDu, HSC3 and CAL-27) treated with PAM. Doxorubicin treatment, serving as positive control, consistently exhibited significant inhibition of cell proliferation across all cell lines, particularly after 48 and 72 h of exposure. In the case of 2 mL PAM, a statistically significant inhibition of cell proliferation, exceeding 50 %, was observed in FaDu (Fig. 2c) and HSC3 (Fig. 2b) cells at all the experimental time points compared to untreated control. Conversely, hGF cells (Fig. 2a) showed only slight inhibition, suggesting a selective cytotoxic effect of PAM towards cancer cell lines. Interestingly, 5 mL PAM treatments showed a generally milder effect on FaDu and HSC3 cancer cells compared to 2 mLPAM on cell proliferation in FaDu, indicating a volume-dependent action of RPMI- and EMEM-PAM (Table S1). Notably, hGF cells remained unaffected by 5 mL PAM treatments. For 2 mL PAM derived from RPMI and EMEM, a time-dependent cytotoxic effect was evident. Indeed, PAM exposed to CAP for 10 and 20 min exerted a greater inhibitory effect on cell proliferation than PAM exposed for 5 min (Table S1).

The CAL-27 tumour cell line (Fig. 2d) demonstrated a relative resistance to DMEM-PAM treatment, particularly when incubated with 5 mL PAM. Only minimal inhibition was observed for the 10 min treatment at 72 h. However, for 2 mL PAM, exposure for 20 min resulted in approximately 50 % inhibition of cell proliferation at 48 and 72 h, while negligible inhibition was observed with shorter exposure times.

### 3.3 The amount of total ROS produced after CAP exposure depends on the medium

**Fig. 3:**
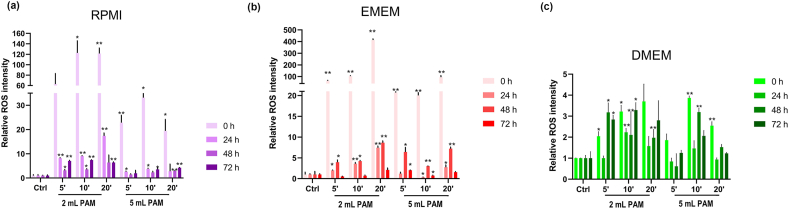
Quantification of total Reactive Oxygen Species (ROS). Histograms represent the relative fluorescence intensity compared to untreated media, set as 1, of (a) RPMI-, (b) EMEM- and (c) DMEM-PAM obtained after activation of two volumes −2 and 5 mL-for 5, 10, and 20 min by exposure to cold atmospheric plasma (CAP). ROS levels were measured at T_0_ and 24, 48, and 72 h after treatment. ∗*p* < 0.05 *vs* 0h Control (Ctrl), ∗∗*p* < 0.01 *vs* 0h Ctrl same experimental time, refer to statistical significance analysed with *t*-test.

The quantification of total ROS in untreated controls and distinct PAM formulations (Fig. 3) reveals that ROS concentrations in 2 mL PAM were generally higher than those generated in 5 mL volumes (initial values at 0 h Fig. 3), reflecting a volume-dependent effect. This trend was particularly evident in RPMI- (Fig. 3a) and EMEM-PAM (Fig. 3b) (Table S2), aligning with the cell proliferation inhibition results observed in the MTS assay. Across nearly all experimental conditions, regardless of the PAM formulation, a significant decrease in ROS concentrations was observed after 24 h of storage at 37 °C in an incubator under conditions simulating cell incubation. Following this initial decrease, ROS levels exhibited minimal variations up to 72h. For RPMI-PAM, ROS concentrations ranged from 1.9 to 12.26 times those of untreated media, whereas EMEM-PAM exhibited a broader range, from 2.0 to 41.3 times. In contrast, DMEM-PAM (Fig. 3c) exhibited modest ROS levels, ranging from 1.8 to 3.8 times those of untreated media, with changes over 72 h that were not linear. A volume- and time-dependent increase in ROS production was evident for 2 mL RPMI- and EMEM-PAM, consistent with the observed inhibitory effects on cell proliferation (Table S3).

#### 3.3.1 In summary

• EMEM-PAM exhibited the highest total ROS levels among all tested conditions.
• RPMI-PAM also showed elevated ROS levels but to a lesser extent compared to EMEM-PAM.
• DMEM-PAM consistently generated the lowest total ROS levels.
• Both RPMI- and EMEM-PAM demonstrated volume- and time-dependent ROS production, with 2 mL volumes and longer CAP treatment durations yielding higher concentrations.

### 3.4 PAM differentially trigger apoptosis induction and cell cycle modifications

To further explore the mechanisms underlying the effects of PAM on HNSCC cells, apoptosis occurrence and cell cycle modifications were analysed 24 h post-treatment. Based on previous results, experimental conditions yielding near-complete cell proliferation inhibition in HSC3 and FaDu 2 mL PAM exposed to CAP for 10 and 20 min, were excluded from these analyses.

**Fig. 4:**
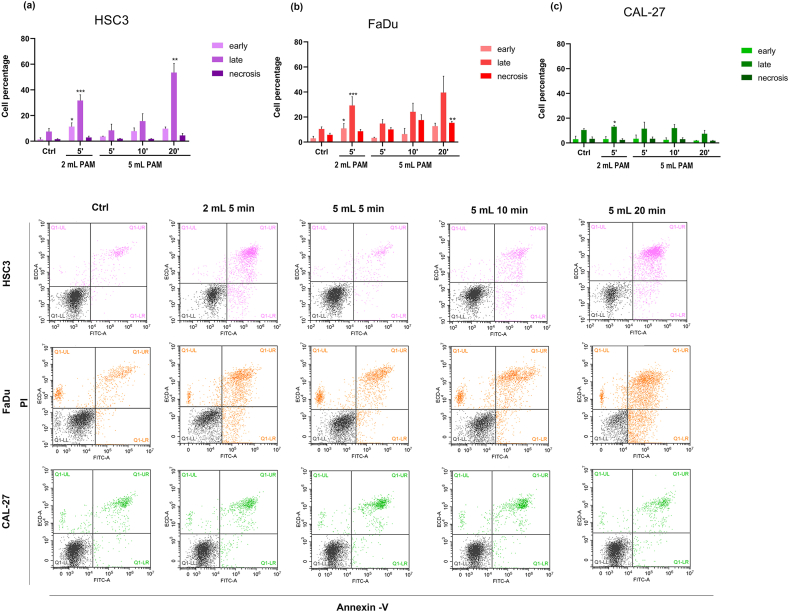
Quantification of apoptosis in three head and neck squamous cell carcinoma (HNSCC) cell lines (a) HSC3, (b) FaDu, and (c) CAL-27. Histograms represent the percentage of cells in early and late apoptosis, and necrosis in cells treated with 2 mL of the respective medium exposed to CAP for 5 min and 5 mL exposed for 5-, 10-, and 20-min. Apoptosis was measured 24 h after the treatment. Representative cytometer dot plots for each cell type are shown (bottom). ∗*p* < 0.05, ∗∗*p* < 0.01 and ∗∗∗*p* < 0.001 *vs* Control (Ctrl) refer to statistical significance analysed with *t*-test.

PAM treatment significantly increased the proportion of cells undergoing late apoptosis in HSC3 (Fig. 4a) and FaDu (Fig. 4b) cell lines. The highest percentages of late apoptotic cells were observed with 2 mL PAM exposed for 5 min and 5 mL PAM exposed for 20 min in both cell lines. Additionally, for 5 mL samples, a clear correlation was observed between longer CAP exposure times and increased apoptosis. In contrast, the CAL-27 cell line demonstrated resistance to PAM treatment, with no significant changes in apoptotic cell populations detected under any tested conditions (Fig. 4c).

**Fig. 5:**
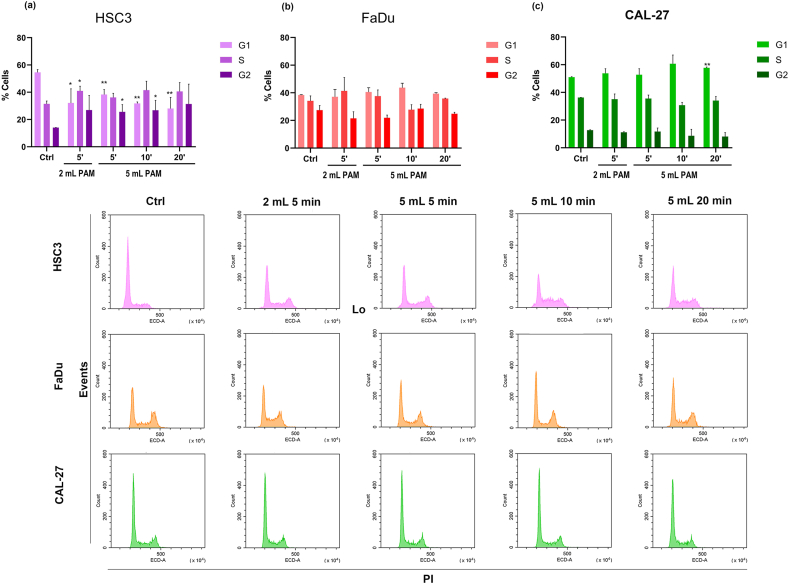
Quantification of cell cycle phases in three head and neck squamous cell carcinoma (HNSCC) cell lines (a) HSC3, (b) FaDu, and (c) CAL-27. Histograms represent the percentage of G_0_/G_1_ (G1), S (S) and G_2_/M (G2) phases in cells treated with 2 mL of the respective medium exposed to CAP for 5 min and 5 mL exposed for 5-, 10-, and 20-min. Cell cycle analysis was performed 24 h after the treatment. Representative cytometer histograms for each cell type are shown (bottom). ∗*p* < 0.05 and ∗∗*p* < 0.01 *vs* Control (Ctrl) refer to statistical significance analysed with t-test.

Cell cycle analyses (Fig. 5) revealed distinct responses among the tested cell lines. HSC3 cells showed a reduction in the percentage of cells in the G_0_/G_1_ phase across all tested conditions following the exposure to RPMI-PAM, with a slight time-dependent trend evident in 5 mL samples (Fig. 5a). Concurrently, an increase in the proportion of cells in the G_2_/M phase was observed across all tested conditions compared to controls. In FaDu cells, a similar trend was present, with a decrease in G_0_/G_1_ phase cells and an increase in G_2_/M phase cells (Fig. 5b); however, these changes lacked a statistical significance. Regarding CAL-27, no changes were detected, except for a mild increase in G_0_/G_1_ phase cells with 5 mL DMEM-PAM exposed to plasma for 20 min (Fig. 5c).

### 3.5 Ultra-structural morphological changes in HNSCC cells exposed to PAM

**Fig. 6:**
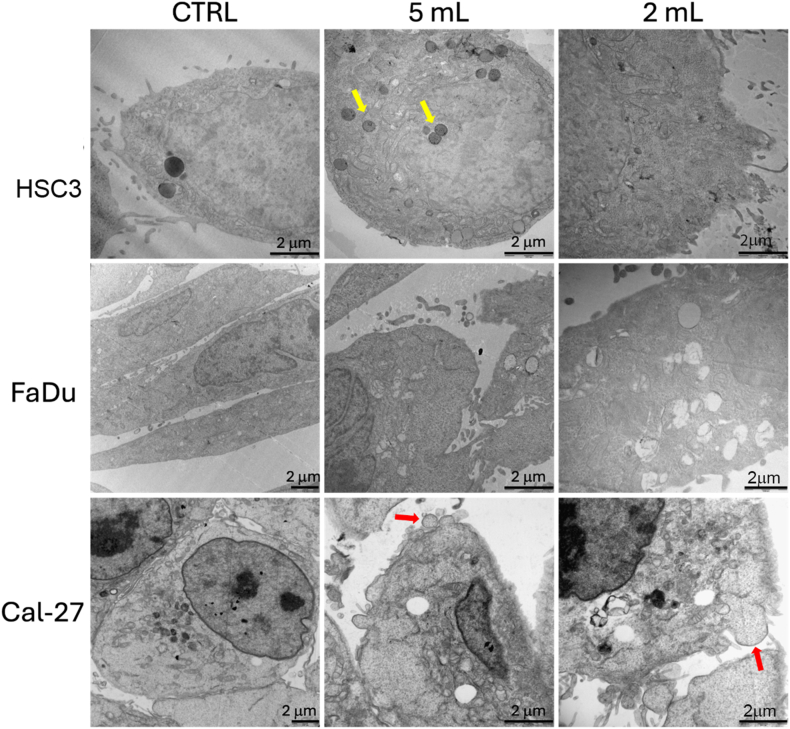
Transmission electron microscopy (TEM) images of HSC3, FaDu and CAL-27 cell lines treated with two volumes - 2 and 5 mL - of the respective medium exposed to CAP for 5 min. Images were taken 24 h after the treatment. Red arrows: plasma membrane blebbing; yellow arrows: lysosomes.

Since the apoptosis assay revealed differences, both in HSC-3 and FaDu cell lines, between 2 mL and 5 mL exposed to CAP for 5 min, for the TEM analyses these two conditions, namely 2 mL-5 min and 5 mL-5 min, were selected to be compared to untreated cells. The choice of these two experimental points seemed to be the most appropriate for electron microscopy analyses, in order to detect morphological features related to apoptosis. 5 mL PAM treated HSC3 show signs of reduced metabolism, such as an increase in lipidic vesicles and electron dense bodies, presumably lysosomes (yellow arrowheads) and minimal mitochondrial damage. In 2 mL PAM treatment, more pronounced effects were detected, including mitochondrial damage, endoplasmatic reticulum (ER) loss, and an increase in the number of free ribosomes in the cytosol. Morphological changes, such as bubbling, were detected and resulted similar to those observed by D. Yan et al. [57]. FaDu cells appeared affected by both 5 mL and 2 mL treatments, which led to mitochondrial damage and dilated ER, indicating significant cellular stress. CAL-27 cells were slightly damaged by PAM treatment and exhibited plasma membrane blebbing (red arrowhead) and a small reduction in mitochondrial density. Fig. 6 highlights these ultrastructural changes, providing further insights into PAM-induced cellular effects across HNSCC cell lines (Fig. 6).

### 3.6 The effects of PAM on cell proliferation depend on the cell culture medium

HSC3 and Fadu cells showed the same high inhibition of proliferation when exposed to their respective media, i.e. RPMI and EMEM, activated with CAP. At the same time, the content of ROS in these PAM was very similar. On the other hand, CAL-27 cell line showed an almost null inhibition of proliferation together with a very low ROS concentration in the medium, DMEM, when exposed to plasma.

**Fig. 7:**
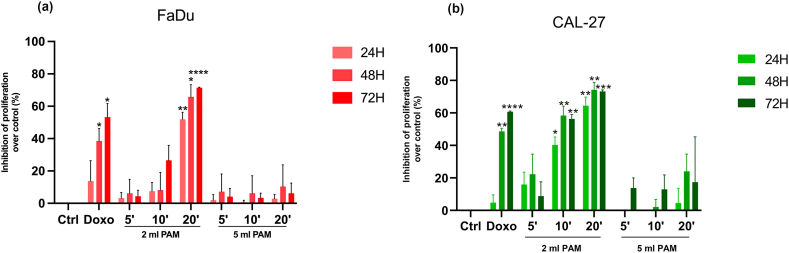
Effects of (a) DMEM-PAM on FaDu and (b) EMEM-PAM on CAL-27 cells. Histograms represent the percentage of proliferation inhibition over the corresponding control cells (Ctrl, expressed as 0 % of inhibition) at 24, 48, and 72 h. Doxorubicin (Doxo) 1 μM treatment was used as positive control. ∗*p* < 0.05, ∗∗*p* < 0.01, ∗∗∗*p* < 0.001, ∗∗∗∗*p* < 0.0001 *vs* Ctrl same experimental time, refer to statistical significance analysed with two-way ANOVA followed by Tukey multiple comparisons test.

In the light of the above observation, CAL-27 cells were compared with one of the other cell line, i.e. FaDU, to understand whether the minimal cytotoxic effect observed after CAL-27 cells were treated with DMEM-PAM was medium-dependent. PAM were swapped between FaDu and CAL-27 cell lines and inhibition of cell proliferation was measured (Fig. 7).

Specifically, FaDu cells were exposed to DMEM-PAM, while CAL-27 cells were treated with EMEM-PAM under the same experimental conditions described in the cell proliferation assays (Fig. 2). The results revealed distinct responses based on the type of PAM. In FaDu, proliferation inhibition is remarkably lower when treated with DMEM-PAM, indicating reduced efficacy in suppressing cell growth (Fig. 7a). Conversely, in CAL-27, exposure to EMEM-PAM resulted in a markedly higher inhibition of proliferation, highlighting its superior effectiveness compared to DMEM-PAM (Fig. 7b). These findings suggest that the inhibitory capacity of PAM is influenced by the specific culture medium used, with DMEM-PAM being less effective in reducing proliferation regardless of the cell line treated.

### 3.7 CAP-treated saline solution anticancer effects and ROS content

To further support the hypothesis that the chemical composition of PAM affects tumour cell proliferation, FaDu and CAL-27 grown in their respective media, were incubated for 2h with PT-SIIITyr solutions activated with CAP fed by air for 5, 10 and 20 min. Previous studies by the co-authors [37,52,58] demonstrated that organic molecules, including phenols, could exert promising anticancer effects, mostly attributed to their pro-oxidant properties and their capacity to generate RONS, such as O_2_^−^, H_2_O_2_, and mixtures of potentially cytotoxic compounds, *in vitro*.

**Fig. 8:**
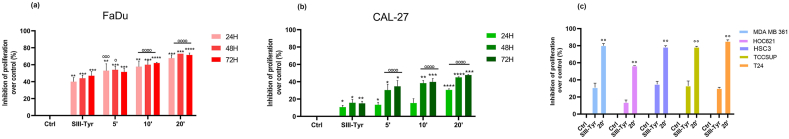
Effects of a saline solution supplement with tyrosine (SIII-Tyr) and activated with CAP fed by air for 5, 10 and 20 min on two head and neck squamous cell carcinoma (HNSCC) cell lines (a) FaDu and (b) CAL-27. Cells were harvested at 24, 48, and 72 h. (c) Effects of a saline solution supplemented with tyrosine (SIII-Tyr) and activated with CAP fed by air for 20 min on two head and neck squamous cell carcinoma cell lines (HOC621 and HSC3), two bladder cancer cell lines (T24 and TCCSUP) and a breast cancer cell line (MDA MB361). Cells were harvested 72hrs after CAP treatment. Histograms represent the percentage of proliferation inhibition over the corresponding control cells (Ctrl, expressed as 0 % of inhibition) ∗*p* < 0.05, ∗∗*p* < 0.01, ∗∗∗*p* < 0.001, *vs* Ctrl same experimental time, o *p* < 0.05 and oooo *p* < 0.0001 vs SIII-Tyr, ^#^*p* < 0.05 and ^####^*p* < 0.0001 *vs* 5 min activation, ^++++^*p* < 0.0001 *vs* 10 min refer to statistical significance analysed with two-way ANOVA followed by Tukey multiple comparisons test.

The results indicate that while FaDu cells (Fig. 8a) show greater sensitivity to CAP-treated SIII-Tyr, both FaDu and Cal-27 cells (Fig. 8b) are susceptible to this treatment. For both cell lines, a clear trend of increased proliferation inhibition was observed with longer plasma treatment durations, with CAP exposure for 20 min achieving the highest inhibitory effect (Fig. 8). Of note, the inhibitory effects of air-fed CAP treatment have also been confirmed in other tumor cell lines, such as HOC621 and HSC3 head and neck cancer cells, as well as in breast and bladder cancer models (Fig. 8c).

**Fig. 9:**
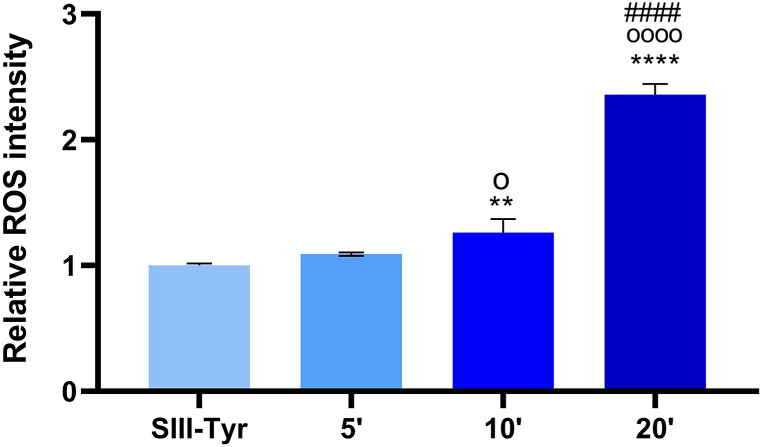
Quantification of total ROS. Histogram represents the relative fluorescence intensity acquired on air plasma treated compared to untreated SIII saline solution supplement with tyrosine (SIII-Tyr), set as 1. ROS levels were measured few minutes before the solutions were administered to the cells. ∗∗*p* < 0.01 and ∗∗∗∗*p* < 0.0001 *vs* Control (SIII-Tyr), ^o^*p* < 0.05 and ^oooo^*p* < 0.0001 *vs* 5 min activation, ^####^*p* < 0.0001 vs 10 min activation, refer to statistical significance analysed with *t*-test.

The quantification of total ROS revealed that SIII saline solution supplemented with tyrosine and subjected to CAP activation for 5, 10, and 20 min exhibited elevated total ROS levels compared to the non-activated control. Furthermore, a clear time-dependent increase in ROS production was observed, with the solution activated for 20 min displaying the highest ROS concentration among all tested conditions (Fig. 9; Table S7).

## 4 Discussion

The worldwide incidence of HNC exceeds half a million cases annually, with up to half of these patients presenting with advanced disease. Surgical resection remains the mainstay of treatment for many HNCs, although radiotherapy, chemotherapy, targeted therapy, and immunotherapy might contribute to an individual patient's treatment plan. Despite these options, prognosis remains suboptimal, especially for advanced-stage tumours. Recent preclinical and clinical trials on direct CAP treatment in HNC have shown promising results, including a) decreased microbial load, odor, and pain with partial tumor suppression [22,23]; b) vascular stimulation or contraction of tumor ulceration accompanied by increased cell apoptosis in CAP-treated tissues [25,59]; c) decreased secretion of inflammatory cytokines [60]. The CAP jet generates a cocktail of reactive species in the gas phase, including RNS like NO and NO₂, and ROS such as OH radicals. Upon dissolving in DW, these reactive species undergo further complex chemical reactions. NO and NO₂ form nitrous acid (HNO₂) and nitric acid (HNO₃) in water, which dissociate into NO₂⁻ and NO₃⁻ ions [61], leading to pH reduction as acidic compounds accumulate. At the same time, the ion concentration increases, raising the water's electrical conductivity [56]. H₂O₂ formed through recombination of OH radicals, is less abundant due to its reactivity and subsequent decomposition in the presence of RNS such as NO and NO_2_ [62]. The overall oxidative capacity of the liquid increases, indicated by an increase in ORP. CAP-induced RONS are known to cause oxidative damage to the cell, resulting in cell death [63]. Despite encouraging results, further investigation into the mechanisms of plasma interaction with biological tissues is essential to refine plasma biotechnology for clinical oncology applications and to mitigate potential side effects. Living tissues exposed to plasma are tipically covered by biological fluids (i.e. saliva, sweat, exudate, blood), making it crucial to study how plasma activated liquids mediate CAP's biological effects. Chemical composition of these fluids after plasma treatment plays a significant role in the observed outcomes alongside other plasma effectors such as electromagnetic field, temperature, UV–vis light. Selective cancer treatments are particularly appealing, as they have the potential to minimize therapy-associated side effects. Although CAP has demonstrated cell-dependent effects, establishing its precision in targeting specific cell types requires thorough characterization of the plasma-treated liquid's chemical composition. The nature of the liquid could indeed dramatically affect the final observed results. In the present study, the influence of the cell culture medium composition on the antitumoral effects of indirect CAP treatment was evaluated, mainly focusing on three HNC cell lines (HSC3, FaDu, and CAL-27).

Cell culture media were used for two reasons: 1) much of the existing literature on plasma processing of liquids for *in vivo* applications is focusesd on cell culture media. Understanding the mechanism of plasma's action on these media can help advance this promising field toward clinical applications; 2) cell culture media are known for their ability to support cell growth and provide essential nutrients. Thus, they serve as an ideal model for simulating biological fluids to better understand the effects of plasma exposure on living tissues.

Additionally, the field of PTL-based anticancer therapy, particularly with FDA approved liquids such as Ringer lactate solution [64], is moving forward, with encouraging results in *in vivo* murine models. However, the investigation of PTL on HNC is in its early stages. The results obtained in the present study indicate that PAM induced a cell-dependent effect, with a notable decrease in cell viability for HSC3 and FaDu cancer cells, which were exposed to media with higher total ROS concentrations. On the other hand, CAL-27 cells appeared less affected by PAM, suggesting some resistance to plasma processing. Indeed, these findings correlate well with the chemical composition of the respective cell culture media. Specifically, plasma treated DMEM exhibited a lower ROS concentration relative to untreated medium in comparison to RPMI and EMEM. Moreover, plasma treated RPMI and EMEM showed a significant decrease in ROS concentration in the first 24 h of storage at 37 °C, whereas DMEM mantained relatively higher ROS levels during this period. Our hypothesis is that these variations in ROS levels can be attributed to the different formulations of the three cell culture media.

**Fig. 10:**
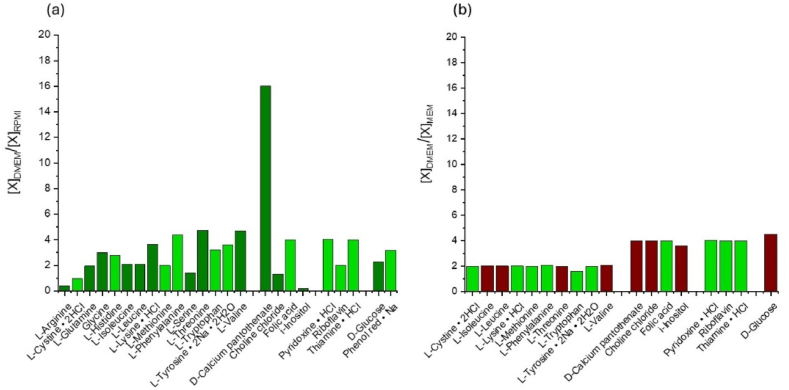
Ratios of concentration of single components of DMEM respect to the same component present in RPMI (a) or EMEM (b). The ratios of organic molecules containing thiol groups or aromatic rings are highlighted in light green.

As shown in Fig. 10, the concentration ratios of organic molecules in the commercial composition of DMEM, RPMI and EMEM differ. DMEM, with respect to the other in particular, is rich in amino acids that contains functional groups or structural elements sensitive to oxidation, such as aromatic and thiol side chains (highlighted in light green in Fig. 10a and b). Many species with aromatic moieties, including L-phenylalanine, L-tryptophan and L-tyrosine, folic acid, pyridoxine, niacinamide, and thiamine are more concentrated in DMEM than in RPMI (Fig. 10a) or EMEM (Fig. 10b). Highly reactive species likely promote the oxidation of these organic molecules, leading to the promoting a scavenging of ROS in the PAM. This scavenging effect could explain why DMEM exhibited lower ROS levels than RPMI and EMEM, following plasma treatment. Additionally, molecules like L-tyrosine known for their pro-oxidant properties could contribute to the overproduction of H_2_O_2_.This, in turn, might counteract the ROS scavenging observed in RPMI and EMEM after 24 h of storage at 37 °C, contributing to the differential effects observed in these media.

Taken together, the results of our study highlight that both the cell type, the cancer type, and composition of the cell culture medium play a crucial role in determining the selectivity and efficacy of CAP treatments. These findings suggest that definitive selectivity of CAP treatment cannot be claimed without first considering these parameters, overlooking them may lead to inaccurate conclusions regarding treatment outcomes.

Our investigation of PAM revealed that different cell culture media generated varying amounts of ROS making it difficult draw definitive conclusions about the sensitivity of different cell types to CAP treatment. To address this limitation, the effect of plasma treatment using the same liquid environment was evaluated. By switching the cell culture media between FaDu and CAL-27, we observed that CAL-27 cells grown in EMEM-PAM, showed significant proliferation inhibition, unlike those grown in DMEM-PAM. The level of inhibition was linearly correlated with the content of total ROS in the PAM formulations. A similar trend was observed in FaDu cells, further confirming that the chemical composition of PAM plays a pivotal role in determining the biological response, as demonstrated also by other studies in literature [27].

In addition to the differences in proliferation inhibition, cell cycle analysis revealed that HSC3 exposed to RPMI-PAM showed a decrease in the percentage of cells in the G_0_/G_1_ phase and an increase in the number of cells in the G_2_/M phase, indicating cell cycle disruption. FaDu cell line shows a similar behaviour, while CAL-27 grown in DMEM-PAM showed no remarkable cell cycle changes likely due to the low ROS levels. Despite the minimal ROS production in DMEM-PAM, a mild damage of the cell membrane, including blebbing, was evident in CAL-27. This observation suggests that even low ROS concentrations can cause cellular damage, potentially initiating laste-stage apoptosis, as indicated by membrane blebbing. These results are in line with the study by Lin et al. [65] where membrane blebbing was attributed to physical effects of direct treatment with radiofrequency electromagnetic modulation, but here it is likely due to chemical effects from ROS, rather than electromagnetic modulation. Blebs are membrane ‘bubbles’ that emerge in the cellular periphery that are formed when the actomyosin cortex, which underlies the plasma membrane, separates from the phospholipid bilayer by contraction or disassembly. The newly formed space fills rapidly with cytoplasm, which increases the volume locally and expands the plasma membrane. The separation of the phospholipid bilayer from the actomyosin cortex should be probably initiated by the peroxidation of lipids by plasma produced ROS in PAM [63]. Blebbing of the plasma membrane is a morphological feature of cells undergoing late-stage apoptosis [67]. The presence of blebbing also in CAL-27 grown in DMEM-PAM shows that, although a low level of ROS is produced in such media, they are enough to initiate a certain cell damage.

Although the results with PAM treatments were promising, the use of cell culture media, which are not clinically approved, calls for a more clinically translational approach. Plasma treated saline solutions, such as SIII (hydrolytic rehydration solution) enriched with tyrosine showed similar inhibition of cell proliferation across all cell types, regardless of the tumor model. Indeed, also bladder and breast cancer cell lines (MDA MB 361, TCCSUP and T24) were sensibile to the PTL cytoxic effects. However, the total ROS levels in PTL were lower compared to those in PAM. These findings support the hypothesis that a combination of ROS and plasma treated organic molecules may enhance the anticancer effect of plasma in a more clinically relevant context [66].

## 5 Conclusion

In conclusion, this study demonstrates that the cytotoxic effects of both PAM and PTL are closely related to the chemical composition of the treated liquid. The influence of cell type was also evident, with CAL-27 showing greater resistance to plasma treated solutions compared to FaDu cells, with an inhibition in proliferation up to 50 % and up to 70 %, respectively. The cytotoxic effects of both PAM and PTL as indirect approaches for cancer treatment was investigated. A tight correlation between the chemical composition of liquids and reduction in cell proliferation, cell cycle alterations and apoptosis occurrence was found. As for the influence of the cell type, CAL-27 proved to be more resistant toPTL (i.e. plasma treated SIII rehydrating infusion solution) compared to FaDu. DMEM reduced the efficacy of plasma treatment, likely due to the presence of ROS scavenging compounds. While differences in the apparent susceptibility of various cancer cell types to plasma treatment were observed, these differences were primarily attributed to the cell culture medium, underscoring the importance of using consistent media when comparing the effects of plasma on different cell types. Ultimately, this study highlights the potential for PTL, including rehydration solutions enriched with tyrosine, to be used as indirect therapeutic approaches for treating HNC. The findings provide valuable insights for future studies aimed at translating plasma medicine into clinical practice, especially in the context of cancer treatment.

As future perspectives, PTL might be tested in combination with other conventional therapies for effective cancer treatment. Specifically, CAP has shown promising potential in enhancing the efficacy of conventional cancer therapies. For instance, CAP's ability to generate RONS could synergize with radiotherapy by increasing oxidative stress in cancer cells. Similarly, the immunomodulatory effects of CAP may enhance the response to immune checkpoint inhibitors, an exciting avenue for further investigation. Previous studies have highlighted the synergistic effects of CAP with chemotherapeutic agents such as gemcitabine [68] and cisplatin [69], suggesting that CAP can amplify the cytotoxic impact of these drugs. Additionally, CAP has been utilized to restore the sensitivity of chemoresistant cancer cells [70], addressing a critical challenge in overcoming drug resistance. Finally, CAP has demonstrated potential in improving the efficacy of immunotherapies, as evidenced in glioblastoma models where plasma exposure enhanced the immune response against tumours [71].

These findings indicate that combining CAP-based treatments with conventional oncological therapies could represent an innovative approach to boosting therapeutic responses and improving clinical outcomes.

## CRediT authorship contribution statement

**Viviana di Giacomo:** Writing – original draft, Investigation, Data curation. **Marwa Balaha:** Investigation, Data curation. **Asia Pece:** Writing – review & editing, Investigation, Data curation. **Ilaria Cela:** Writing – review & editing, Data curation. **Gianluca Fulgenzi:** Investigation, Data curation. **Giovanna Orsini:** Investigation, Data curation. **Tatiana Spadoni:** Investigation, Data curation. **Tirtha Raj Acharya:** Writing – original draft, Data curation, Formal analysis. **Nagendra Kumar Kaushik:** Resources, Funding acquisition, Formal analysis. **Eun Ha Choi:** Resources. **Monica Rapino:** Investigation. **Mariangela Mazzone:** Investigation, Data curation. **Gabriella Mincione:** Writing – review & editing, Investigation, Data curation. **Gianluca Sala:** Writing – review & editing. **Eloisa Sardella:** Writing – review & editing, Resources. **Vittoria Perrotti:** Writing – review & editing, Project administration, Funding acquisition, Conceptualization.

## Data availability statement

Data will be made available on request.

## Ethics statement

Review and approval by an ethics committee were not needed for this study because this study did not involve animal or human experiments.

## Funding statement

This research was funded by NextGenerationEU - MUR, Fondo Promozione e Sviluppo, DM 737/2021 - Project title: COld atmospheric plasma therapy to target head and neck Tumours by A multImodaL approach; Acronym: Cocktail; 10.13039/501100002446CUP number: D75F21003210001; and funded by PRINPNRR 2022-P2022F4P8P-Bi-functioNal plasma-treated solutions as a new thErapeutiC Tool for cAnceR (NECTAR). GS is funded by Fondazione-AIRC [IG 2021 id 25696]. This study was also supported by the 10.13039/501100001321National Research Foundation (10.13039/501100003725NRF) of Korea, funded by the Korean government 2021R1A6A1A03038785.

## Declaration of competing interest

The authors declare that they have no known competing financial interests or personal relationships that could have appeared to influence the work reported in this paper.
