# Supplementary material for: Human head and neck cancer cell lines response to cold atmospheric plasma activated media is affected by the chemistry of culture media
Source: Heliyon. 2024 Dec 25;11(1):e41458. doi: 10.1016/j.heliyon.2024.e41458 (PMC11757765; doi:10.1016/j.heliyon.2024.e41458)
Supplement: Multimedia component 1 [file mmc1.docx]

Supplementary material

**Human head and neck cancer cell lines response to cold atmospheric plasma activated media is affected by the chemistry of culture media**

Viviana di Giacomo^1,2^, Marwa Balaha^3,4^, Ilaria Cela^3,5^, Gianluca Fulgenzi^6^, Giovanna Orsini^7^, Tatiana Spadoni^6^, Tirtha Raj Acharya^8^, Nagendra Kumar Kaushik^8^, Eun Ha Choi^8^, Monica Rapino^9^, Mariangela Mazzone^3^, Gabriella Mincione^3^, Gianluca Sala^3,5^, Eloisa Sardella^10^, Vittoria Perrotti^2,3^*

1 Department of Pharmacy, “G. d’Annunzio” University of Chieti-Pescara, Chieti, Italy; [viviana.digiacomo@unich.it](mailto:viviana.digiacomo@unich.it) (V.d.G.);

2 UdA-TechLab, Research Center, “G. d’Annunzio” University of Chieti-Pescara, Chieti, Italy;

3 Department of Innovative Technologies in Medicine & Dentistry, “G. d’Annunzio” University of Chieti-Pescara, Chieti, Italy; [marwa.balaha@unich.it](mailto:marwa.balaha@unich.it) (M.B.); [ilaria.cela@unich.it](mailto:ilaria.cela@unich.it) (I.C.); [mariangela.mazzone@unich.it](mailto:mariangela.mazzone@unich.it) (M.M.); [gabriella.mincione@unich.it](mailto:gabriella.mincione@unich.it) (G.M.); [gianluca.sala@unich.it](mailto:gianluca.sala@unich.it) (G.S.); [vittoria.perrotti@unich.it](mailto:vittoria.perrotti@unich.it) (V.P.);

4 Department of Pharmaceutical Chemistry, Faculty of Pharmacy, Kafrelsheikh University, 33516 Kafr El Sheikh, Egypt;

5 Center for Advanced Studies and Technology (CAST), “G. d’Annunzio” University of Chieti-Pescara, Chieti, Italy;

6 Department of Clinical and Molecular Sciences, Polytechnic University of Marche, Ancona, Italy; [g.fulgenzi@staff.univpm.it](mailto:g.fulgenzi@staff.univpm.it) (G.F.); [t.spadoni@univpm.it](mailto:t.spadoni@univpm.it) (T.S.);

7 Department of Clinical Sciences and Stomatology (DISCO), Polytechnic University of Marche, Ancona, Italy. [g.orsini@univpm.it](mailto:g.orsini@univpm.it)

8 Plasma Bioscience Research Center, Department of Electrical and Biological Physics, Kwangwoon University, Seoul 01897, South Korea; kaushik.nagendra@kw.ac.kr (N.K.); [ehchoi@kw.ac.kr](mailto:ehchoi@kw.ac.kr) (E.C.);

9 Genetic Molecular Institute of CNR, Unit of Chieti, “G. d’Annunzio” University of Chieti-Pescara, Chieti, Italy; [monica.rapino@unich.it](mailto:monica.rapino@unich.it) (M.R.).

10 CNR- Istituto di Nanotecnologia (CNR-NANOTEC) UoS Bari, c/o Dipartimento di Chimica, Università degli Studi di Bari Aldo Moro, via Orabona, 4, 70126 Bari, Italy; [eloisa.sardella@cnr.it](mailto:eloisa.sardella@cnr.it)

**Table S1.** Time- and volume-dependent statistical significance of inhibitory effects of 2 mL and 5 mL PAM on three tumoral HNC cell lines (HSC3, FaDu, and CAL-27) proliferation rates. *p<0.05, **p<0.01, ***p<0.001, ns, not significant.

| **HSC3** | **Time-dependence** | **24h** | **48h** | **72h** |
| --- | --- | --- | --- | --- |
|  | 2 mL 5’ vs 2 mL 10’ | **ns** | **ns** | **ns** |
|  | 2 mL 5’ vs 2 mL 20’ | **ns** | **ns** | **ns** |
|  | 2 mL 10’ vs 2 mL 20’ | **ns** | **ns** | **ns** |
|  | 5 mL 5’ vs 5 mL 10’ | **ns** | **ns** | **ns** |
|  | 5 mL 5’ vs 5 mL 20’ | ***** | **ns** | ***** |
|  | 5 mL 10’ vs 5 mL 20’ | **ns** | **ns** | **ns** |
|  | **Volume-dependence** | **24h** | **48h** | **72h** |
|  | 2 mL 5’ vs 5 mL 5’ | ***** | ***** | ***** |
|  | 2 mL 10’ vs 5 mL 10’ | ***** | **ns** | **ns** |
|  | 2 mL 20’ vs 5 mL 20’ | **ns** | **ns** | **ns** |
| **FaDu** | **Time-dependence** | **24h** | **48h** | **72h** |
|  | 2 mL 5’ vs 2 mL 10’ | **ns** | **ns** | **ns** |
|  | 2 mL 5’ vs 2 mL 20’ | **ns** | **ns** | **ns** |
|  | 2 mL 10’ vs 2 mL 20’ | **ns** | **ns** | **ns** |
|  | 5 mL 5’ vs 5 mL 10’ | **ns** | **ns** | **ns** |
|  | 5 mL 5’ vs 5 mL 20’ | ***** | ****** | ***** |
|  | 5 mL 10’ vs 5 mL 20’ | **ns** | **ns** | **ns** |
|  | **Volume-dependence** | **24h** | **48h** | **72h** |
|  | 2 mL 5’ vs 5 mL 5’ | ****** | ****** | ******* |
|  | 2 mL 10’ vs 5 mL 10’ | **ns** | **ns** | **ns** |
|  | 2 mL 20’ vs 5 mL 20’ | **ns** | **ns** | **ns** |
| **CAL-27** | **Time-dependence** | **24h** | **48h** | **72h** |
|  | 2 mL 5’ vs 2 mL 10’ | **ns** | **ns** | **ns** |
|  | 2 mL 5’ vs 2 mL 20’ | **ns** | ****** | ***** |
|  | 2 mL 10’ vs 2 mL 20’ | **ns** | **ns** | **ns** |
|  | 5 mL 5’ vs 5 mL 10’ | **ns** | **ns** | **ns** |
|  | 5 mL 5’ vs 5 mL 20’ | **ns** | **ns** | **ns** |
|  | 5 mL 10’ vs 5 mL 20’ | **ns** | **ns** | **ns** |
|  | **Volume-dependence** | **24h** | **48h** | **72h** |
|  | 2 mL 5’ vs 5 mL 5’ | **ns** | **ns** | **ns** |
|  | 2 mL 10’ vs 5 mL 10’ | **ns** | **ns** | **ns** |
|  | 2 mL 20’ vs 5 mL 20’ | **ns** | ***** | ***** |

**Table S2:** Volume-dependent statistical significance of total Reactive Oxygen Species (ROS) in RPMI-, EMEM- and DMEM-PAM obtained after activation of two volumes -2 and 5 mL- for 5, 10, and 20 minutes by exposure to cold atmospheric plasma (CAP). *p<0.05, **p<0.01, ns, not significant.

|  | **0 h, 5 mL** | | | **24 h, 5 mL** | | | **48 h, 5 mL** | | | **72 h, 5 mL** | | |
| --- | --- | --- | --- | --- | --- | --- | --- | --- | --- | --- | --- | --- |
| RPMI | **5**  **min** | **10**  **min** | **20**  **min** | **5**  **min** | **10**  **min** | **20**  **min** | **5**  **min** | **10**  **min** | **20**  **min** | **5**  **min** | **10**  **min** | **20**  **min** |
| **2ml 5min** | * |  |  | ** |  |  | * |  |  | * |  |  |
| **2ml 10min** |  | * |  |  | ** |  |  | ns |  |  | * |  |
| **2ml 20min** |  |  | ** |  |  | * |  |  | ns |  |  | ** |

|  | **0 h, 5 mL** | | | **24 h, 5 mL** | | | **48 h, 5 mL** | | | **72 h, 5 mL** | | |
| --- | --- | --- | --- | --- | --- | --- | --- | --- | --- | --- | --- | --- |
| EMEM | **5**  **min** | **10**  **min** | **20**  **min** | **5**  **min** | **10**  **min** | **20**  **min** | **5**  **min** | **10**  **min** | **20**  **min** | **5**  **min** | **10**  **min** | **20**  **min** |
| **2ml 5min** | ** |  |  | ns |  |  | ns |  |  | ** |  |  |
| **2ml 10min** |  | ** |  |  | * |  |  | * |  |  | * |  |
| **2ml 20min** |  |  | ** |  |  | * |  |  | ns |  |  | ns |

|  | **0 h, 5 mL** | | | **24 h, 5 mL** | | | **48 h, 5 mL** | | | **72 h, 5 mL** | | |
| --- | --- | --- | --- | --- | --- | --- | --- | --- | --- | --- | --- | --- |
| DMEM | **5**  **min** | **10**  **min** | **20**  **min** | **5**  **min** | **10**  **min** | **20**  **min** | **5**  **min** | **10**  **min** | **20**  **min** | **5**  **min** | **10**  **min** | **20**  **min** |
| **2ml 5min** | ns |  |  | ns |  |  | * |  |  | * |  |  |
| **2ml 10min** |  | ns |  |  | ns |  |  | * |  |  | ns |  |
| **2ml 20min** |  |  | ns |  |  | * |  |  | ** |  |  | * |

**Table S3:** Time-dependent statistical significance of total Reactive Oxygen Species (ROS) in RPMI-, EMEM- and DMEM-PAM obtained after activation of 2 mL for 5, 10, and 20 minutes by exposure to cold atmospheric plasma (CAP). *p<0.05, **p<0.01, ns, not significant.

| RPMI | **0 h, 2 mL** | | **24 h, 2 mL** | | **48 h, 2 mL** | | **72 h, 2 mL** | |
| --- | --- | --- | --- | --- | --- | --- | --- | --- |
|  | **5min** | **10min** | **5min** | **10min** | **5min** | **10min** | **5min** | **10min** |
| **2ml 10min** | * |  | * |  | ns |  | ns |  |
| **2ml 20min** | * | ns | ** | * | ns | ns | ns | * |

| EMEM | **0 h, 2 mL** | | **24 h, 2 mL** | | **48 h, 2 mL** | | **72 h, 2 mL** | |
| --- | --- | --- | --- | --- | --- | --- | --- | --- |
|  | **5min** | **10min** | **5min** | **10min** | **5min** | **10min** | **5min** | **10min** |
| **2ml 10min** | ** |  | * |  | ns |  | ns |  |
| **2ml 20min** | ** | ** | * | * | * | ** | * | ns |

| DMEM | **0 h, 2 mL** | | **24 h, 2 mL** | | **48 h, 2 mL** | | **72 h, 2 mL** | |
| --- | --- | --- | --- | --- | --- | --- | --- | --- |
|  | **5min** | **10min** | **5min** | **10min** | **5min** | **10min** | **5min** | **10min** |
| **2ml 10min** | * |  | ** |  | * |  | ns |  |
| **2ml 20min** | ns | ns | * | ns | * | ns | ns | ns |

**Table S4.** Quantification of total Reactive Oxygen Species (ROS). The relative fluorescence intensity compared to untreated media, set as 1, of RPMI-PAM obtained after activation of two volumes -2 and 5 mL- for 5, 10, and 20 min by exposure to cold atmospheric plasma (CAP).

|  | 0 h | 24 h | 48 h | 72 h |
| --- | --- | --- | --- | --- |
| Ctrl | 1±0.37 | 1±0.41 | 1±0.14 | 1±0.29 |
| 2ml 5min | 62.78±20.33 | 8.19±0.19 | 3.14±0.36 | 7.06±0.23 |
| 2ml 10min | 122.65±23.06 | 9.04±0.17 | 3.51±0.35 | 7.45±0.09 |
| 2ml 20min | 121.46±10.44 | 17.37±1.18 | 6.39±3.09 | 6.4±0.27 |
| 5ml 5min | 22.84±3.04 | 2.61±0.25 | 1.41±0.36 | 1.95±1.23 |
| 5ml 10min | 33.17±5.06 | 3.75±0.28 | 2.29±0.50 | 3.56±0.58 |
| 5ml 20min | 19.43±4.76 | 2.78±2.93 | 2.94±0.07 | 4.26±0.07 |

**Table S5.** Quantification of total Reactive Oxygen Species (ROS). The relative fluorescence intensity compared to untreated media, set as 1, of EMEM-PAM obtained after activation of two volumes -2 and 5 mL- for 5, 10, and 20 min by exposure to cold atmospheric plasma (CAP).

|  | 0 h | 24 h | 48 h | 72 h |
| --- | --- | --- | --- | --- |
| Ctrl | 1±0.54 | 1±0.17 | 1±0.72 | 1±0.16 |
| 2ml 5min | 64.35±3.17 | 1.97±0.09 | 3.93±0.55 | 0.52±0.04 |
| 2ml 10min | 105.64±2.33 | 3.58±0.29 | 4.21±0.17 | 0.69±0.16 |
| 2ml 20min | 413.21±9.61 | 7.42±0.40 | 8.63±0.42 | 2.08±0.47 |
| 5ml 5min | 20.72±0.74 | 1.20±0.33 | 6.42±1.11 | 2.03±0.03 |
| 5ml 10min | 20.04±0.65 | 0.32±0.03 | 3.01±0.02 | 0.73±0.09 |
| 5ml 20min | 95.16±13.02 | 2.74±0.53 | 7.21±0.38 | 1.55±0.14 |

**Table S6.** Quantification of total Reactive Oxygen Species (ROS). The relative fluorescence intensity compared to untreated media, set as 1, of DMEM-PAM obtained after activation of two volumes -2 and 5 mL- for 5, 10, and 20 min by exposure to cold atmospheric plasma (CAP).

|  | 0 h | 24 h | 48 h | 72 h |
| --- | --- | --- | --- | --- |
| Ctrl | 1.00±0.01 | 1.00±0.01 | 1.00±0.13 | 1.00±0.31 |
| 2ml 5min | 2.04±0.21 | 1.00±0.10 | 3.18±0.44 | 2.84±0.19 |
| 2ml 10min | 3.21±0.29 | 2.23±0.18 | 2.10±1.10 | 3.29±0.35 |
| 2ml 20min | 3.70±0.82 | 1.57±0.45 | 1.97±0.39 | 2.80±0.93 |
| 5ml 5min | 1.85±0.29 | 0.84±0.16 | 0.61±0.59 | 1.25±0.12 |
| 5ml 10min | 3.86±0.09 | 1.46±0.36 | 3.19±0.19 | 2.06±0.25 |
| 5ml 20min | 2.54±0.18 | 0.93±0.08 | 1.52±0.12 | 1.23±0.04 |

**Table S7.** Quantification of total Reactive Oxygen Species (ROS). The relative fluorescence intensity compared to untreated SIII saline solution supplement with tyrosin (SIII-Tyr), set as 1. ROS levels were measured when the solutions were administered to the cells.

| SIII-Tyr | 1±0.01 |
| --- | --- |
| 5min | 1.09±0.01 |
| 10min | 1.26±0.10 |
| 20min | 2.36±0.08 |
